# The Future of Foundation Machine Learning Potentials and DFT in Homogeneous Catalysis: Competition or Synergy?

**DOI:** 10.1002/chem.71022

**Published:** 2026-04-18

**Authors:** Maxime Ferrer, Julen Munarriz, Thijs Stuyver, Ruben Laplaza

**Affiliations:** ^1^ Ecole Nationale Supérieure de Chimie de Paris, CNRS i‐CLeHS Paris France; ^2^ Departamento de Química Física and Instituto de Biocomputación y Física de Sistemas Complejos Universidad de Zaragoza Zaragoza Spain; ^3^ IIQ, Instituto de Investigaciones Químicas (CSIC‐Universidad de Sevilla) Consejo Superior de Investigaciones Científicas Seville Spain

**Keywords:** catalysis, computational chemistry, DFT, homogeneous catalysis, ML

## Abstract

While DFT is the computational method of choice for mechanistic insight in homogeneous catalysis, the recent rise of foundation‐level machine learning interatomic potentials (MLIPs) invites reconsideration: are we approaching competition, or a deeper synergy? These pretrained, fast surrogates are able to map reaction space, sample conformers, and flag likely transition states, potentially displacing routine low‐level DFT. Yet their reliability hinges on calibrated uncertainty, transferability across ligand and oxidation‐state manifolds, and faithful treatment of long‐range polarization, solvation, and open‐shell or multireference character. We argue that the near future will likely be contested: MLIPs will handle everyday exploratory tasks, while DFT and higher‐level methods will anchor electronic effects, validate high‐stakes predictions, and resolve edge cases. If supported by FAIR catalysis datasets, standardized workflows, and robust error quantification, the two approaches will coevolve, enabling scalable, predictive discovery without sacrificing rigor or interpretability.

## Graphical abstract

Machine‐learning potentials are increasingly taking on the exploratory tasks of homogeneous catalysis, enabling rapid conformer sampling and reaction‐space mapping. However, when selectivity depends on subtle electronic effects, electronic‐structure methods remain essential. Rather than replacement, the future likely lies in a layered predictive framework where computational speed and electronic rigor evolve together.

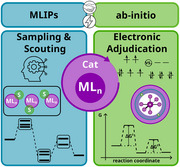

## 1 Introduction

Density functional theory (DFT) remains the workhorse for mechanistic analysis and rational design in homogeneous catalysis [1, 2, 3], enabling free‐energy profile computations, selectivity rationalizations, and microkinetic models that bridge computation and experiment [4, 5]. In organometallic chemistry, these capabilities often guide hypothesis generation and reaction optimization. However, longstanding limitations still constrain scope and reliability. Results depend on the functional and basis set, finite‐temperature and solvation corrections remain fragile, open‐shell and multireference regimes remain challenging, and the computational cost rises steeply with system size and conformational complexity [6, 7, 8, 9, 10, 11, 12]. These issues become acute when competing spin manifolds or subtle electronic effects control selectivity, which motivates both wavefunction benchmarks for accuracy and approximate semiempirical methods for throughput [13, 14, 15, 16, 17].

Foundation‐level machine‐learning interatomic potentials (foundation MLIPs) add a new axis to this landscape [18, 19, 20]. By this we mean models trained on broad, heterogeneous datasets, that aim to capture transferable patterns of bonding and reactivity, in contrast to traditional MLIPs constructed for a specific catalyst or reaction. Trained on large quantum‐chemical datasets, modern MLIPs often approach DFT‐level energies and forces at much lower computational cost, although performance varies across chemical space and zero‐shot transfer remains uneven [21, 22, 23, 24]. It is also important to note that semiempirical methods are an active area of research and may offer competitive gains in both speed and accuracy [25, 26, 27], sometimes incorporating core machine‐learning tools into their parameterization as well [28, 29]. Here, however, we use the term MLIPs specifically to denote models that do not include any explicit quantum‐chemical equations, even in approximate form.

For computational catalysis, the implication is straightforward. If a given foundation MLIP performs reliably within a target domain, the main bottleneck shifts from electronic‐structure evaluation to large‐scale sampling and exploration. Conformational analysis, reaction‐space mapping, and transition‐state candidate identification are areas where MLIPs could replace routine DFT steps [30, 31, 32]. On the other hand, synergy remains unavoidable wherever electronic structure controls the outcome. Selectivity‐determining barriers, spin crossings, charge transfer, and multireference character require diagnostics and controlled accuracy from DFT and higher‐level methods. A further complication is that most foundation MLIPs are trained on DFT data, and therefore systematic DFT biases may unpredictably propagate into ML predictions. Reliability depends on calibrated uncertainty and explicit escalation pathways [33, 34, 35].

Whether this emerging division of labor leads to the displacement of routine DFT or to co‐evolution with electronic‐structure methods will depend on usability, data quality, physics coverage, and software infrastructure. Catalysis‐relevant training and benchmark sets must include transition‐metal chemistry with explicit charge and spin labeling, as well as reactive, off‐equilibrium configurations that extend beyond current FAIR efforts. In parallel, model developments that better capture long‐range electrostatics, polarization, and solvation are essential for realistic solution‐phase catalysis [36, 37]. Finally, broad adoption will require interfaces and standardized workflows that integrate uncertainty, escalation, and reproducibility, analogous to the software ecosystems that underpin modern quantum chemistry [38, 39].

**FIGURE 1:**
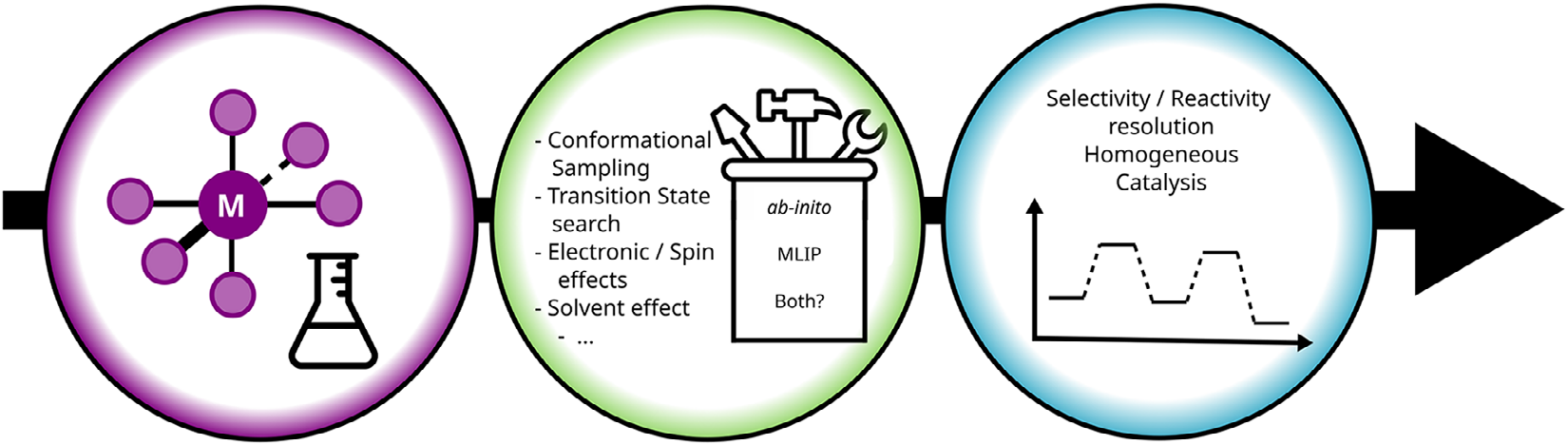
Conceptual landscape of competition and synergy between foundation machine‐learning potentials and ab initio methods in homogeneous catalysis.

In this perspective, we take a speculative, forward‐looking stance. Our goal is not to declare a winner between MLIPs and DFT, but to outline the conditions under which competition becomes credible and the circumstances in which tight coupling is indispensable. The sections that follow examine how reliability should be quantified and managed, the present limitations and bottlenecks, how catalysis‐specific datasets and benchmarks must evolve, and how model outputs may interface with microkinetic modeling and experiment to yield trustworthy, actionable predictions (Figure 1).

## 2 The Arrival of Foundation‐Level ML Potentials: Reality Check

For many catalysis practitioners, “machine‐learning potentials” refers to bespoke models trained for a single system, such as one catalyst, one solvent, and one reaction, with substantial data generation and expert effort [36, 41, 42, 43, 44]. Foundation MLIPs change this picture [18, 19, 20, 23]. Rather than training end to end for a narrow target, a foundation MLIP is pretrained on an extremely large and heterogeneous set of quantum‐mechanical reference calculations. It learns reusable representations of chemical environments and their energies and forces. The practical promise is zero‐shot use, or light adaptation, on new systems by leveraging patterns learned across molecules, materials, and catalytic motifs including bond formation and breaking.

**FIGURE 2:**
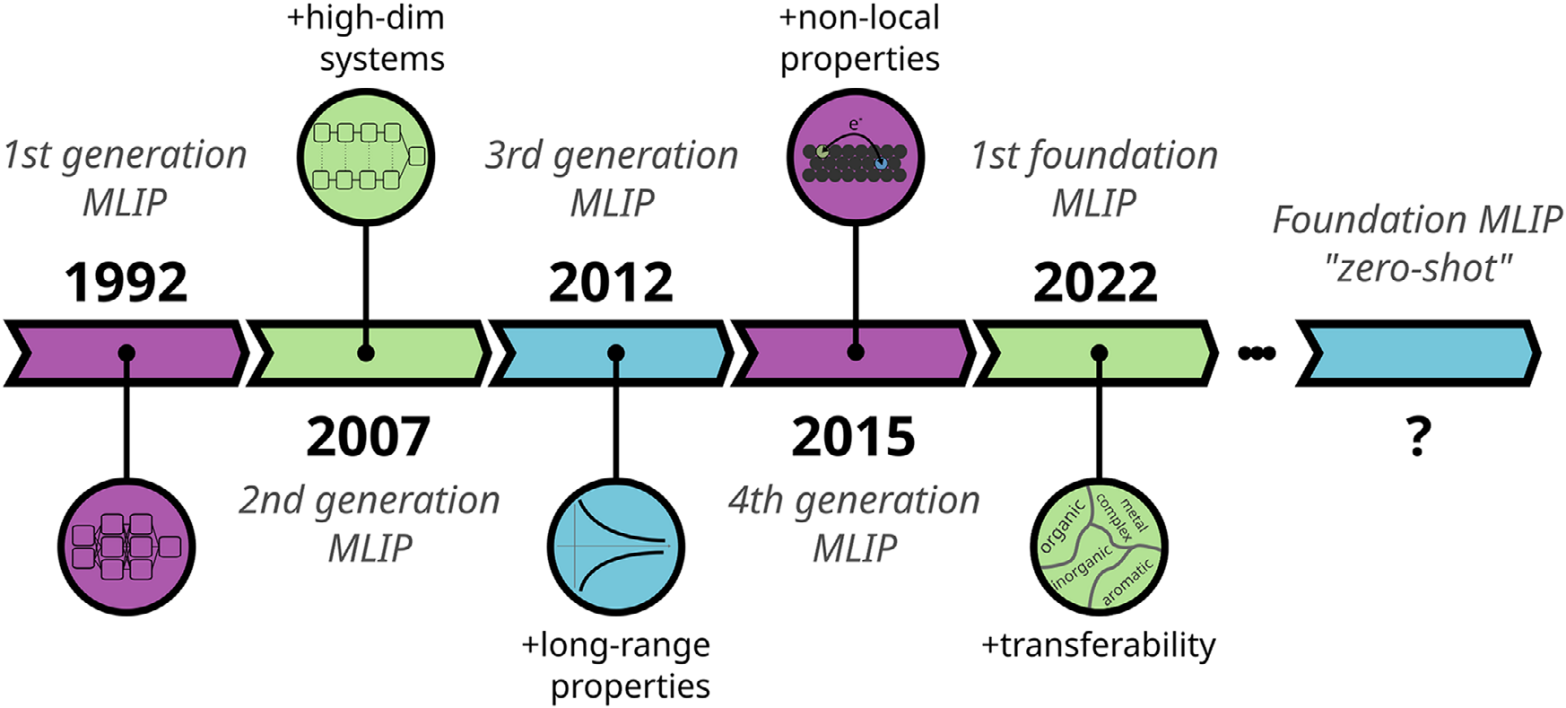
Chronological evolution of machine‐learning interatomic potentials, inspired by the MLIP classification proposed by Behler [40].

A useful analogy is a pretrained large language model. Pretraining does not guarantee perfect performance on every new topic, yet it provides a strong prior that transfers across tasks. In atomistic modeling, that prior is an efficient mapping from structure to energies and forces that supports geometry optimization, molecular dynamics, and large‐scale sampling at costs far below routine DFT. What distinguishes foundation MLIPs from earlier generations is not the idea of learning a potential energy surface, but the scale and diversity of training data and the resulting expectation of broader transferability (Figure 2) [40, 45, 46].

Recent releases make the foundation idea tangible. The Open Molecules 2025 (OMol25) database provides consistent, hybrid‐DFT‐quality energies and forces for more than $10^{8}$ systems, and it stores total charge and spin multiplicity [47, 48]. OMol25 is both large and diverse, and it includes substantial non‐equilibrium coverage rather than only optimized minima [47, 48]. This matters because potentials used in dynamics must remain stable not only near equilibrium, but also for distorted configurations and reactive regions that trajectories explore.

Building on multi‐domain data, Meta's Universal Models for Atoms (UMA) exemplify a foundation MLIP trained at unprecedented scale across molecules, materials, and catalysis [23]. The aim is to amortize data generation and model development by training on hundreds of millions of distinct 3D structures from multiple domains, so that one model supports tasks that previously relied on specialized MLIPs [23]. A foundation MLIP is not a chemistry oracle, but a broadly pretrained surrogate for DFT energies and forces that aims to be generally usable.

This reality check has two parts. First, “DFT‐level” claims for foundation MLIPs usually refer to energies and forces on benchmark test sets. This often suffices for high‐throughput conformer sampling, reaction‐space exploration, and transition‐state candidate generation, yet it does not imply chemically accurate barriers, selectivities, or robust behavior under large distribution shifts, such as modeling novel chemical reactions [18, 23].

Second, transfer is conditional. Homogeneous catalysis spans changes in oxidation state, spin, coordination topology, and solvation environment, so zero‐shot reliability depends on whether these motifs appear in pretraining and whether the model captures the physics needed for smooth extrapolation [23, 47]. This motivates a guiding principle used throughout this Perspective: foundation MLIPs are best treated as reusable priors that replace routine exploratory steps in well‐known chemical domains, while DFT and higher‐level methods remain essential when electronic structure is decisive or when uncertainty indicates extrapolation. Therefore, the challenge for practitioners in the near future is judiciously navigating between these two approaches.

## 3 Where Competition is Credible

The strongest case for competition between MLIPs and DFT in homogeneous catalysis is the replacement of routine scouting, where cost is dominated by sampling rather than single‐point fidelity. Foundation MLIPs raise the throughput of large‐scale conformer enumeration, reaction‐space mapping, and transition‐state proposal generation to levels that remain prohibitive for modest DFT settings [23, 47, 48]. Additionally, MLIPs running on GPUs benefit from large‐scale simulations (in terms of atoms) or trivially parallel tasks, where the full memory of the chip is used during runtime. In this breadth‐first regime, the key question is not universal accuracy, but rather whether the model ranks and prioritizes a small set of chemically decisive structures well enough for later DFT adjudication.

Credibility requires an explicit applicability domain defined by elements, key ligand motifs, and relevant charge and spin states. In practice, the first step is domain declaration rather than geometry optimization. The declaration states which metals and ligands, charge and spin states, solvent motifs (if any), and which reference DFT level the workflow aims to emulate. This mirrors standard practice in DFT, where method choice depends on the problem class [2, 6], albeit with additional importance.

Within a declared domain, three task types support ML‐first replacement of routine DFT or semiempirical methods. Conformer and speciation sampling benefit because flexible ligands, coordination isomers, ion‐pairing motifs, and weak encounter complexes demand ensemble coverage. When the goal is low‐lying structures and relative populations, rather than sub‐kcal ${\mathrm{mol}}^{-1}$ barrier differences, MLIPs replace most scouting optimizations and enable sampling workflows that would otherwise be truncated [30, 31, 32, 49]. Reaction–space mapping and pathway triage benefit because screening plausible routes, such as alternative insertions, ligand exchange, and variants of β‐H elimination, often requires dozens of optimizations. Transition‐state optimization requires locating saddle‐like geometries and reaction coordinates in the potential energy surface, tasks that are typically hampered by the high cost of computing first and second derivatives of the energy with DFT. Owing to their differentiability, MLIPs provide hessians at a low cost [50, 51], which open the door to thorough saddle point optimization algorithms and quick, effective exploration [52].

Operationally, MLIP outputs are best treated as high‐coverage proposals with safeguards rather than direct replacements for DFT. Trust is highest within the declared domain and after basic stability checks during short optimizations or short dynamics. Trust improves when these runs show no force spikes, no systematic energy drift, no unphysical rearrangements, and no persistent nonconvergence. The choice must match the task. Substitution is most defensible for ranking, sampling, and proposal generation, not for mechanistic claims that hinge on small energy gaps. A calibration and escalation plan is equally important: uncertainty should be monitored, even through practical proxies, and flagged structures should move to DFT automatically rather than through ad hoc judgment [34, 35].

Importantly, benchmarking should emulate the workflow at hand, not a static test set. A claim of replacing a routine DFT protocol is falsifiable if the MLIP reproduces the qualitative behavior of that protocol for the structures and distortions produced in use. Validation benefits from task‐specific designs, such as conformer ranking, coordination rearrangements, and stability near transition structures, with splits and perturbations that mimic catalytic workflows rather than random holdouts [53]. It must be acknowledged that benchmark construction is not neutral. Curated sets often overrepresent stable, easy structures and understate real‐world error. Stress tests that target difficult regions, for example, configurations where methods disagree most, provide a more meaningful safety check for both DFT and MLIP deployment [54, 55].

The practical takeaway is that competition is most credible when MLIPs replace the expensive, repetitive parts of a catalysis workflow, namely scouting, sampling, and proposal generation, while DFT and higher level methods are reserved for electronically decisive points. The goal is fewer DFT calculations per mechanistic conclusion, without lowering standards for validation and interpretability.

## 4 Where Synergy is Inevitable

**FIGURE 3:**
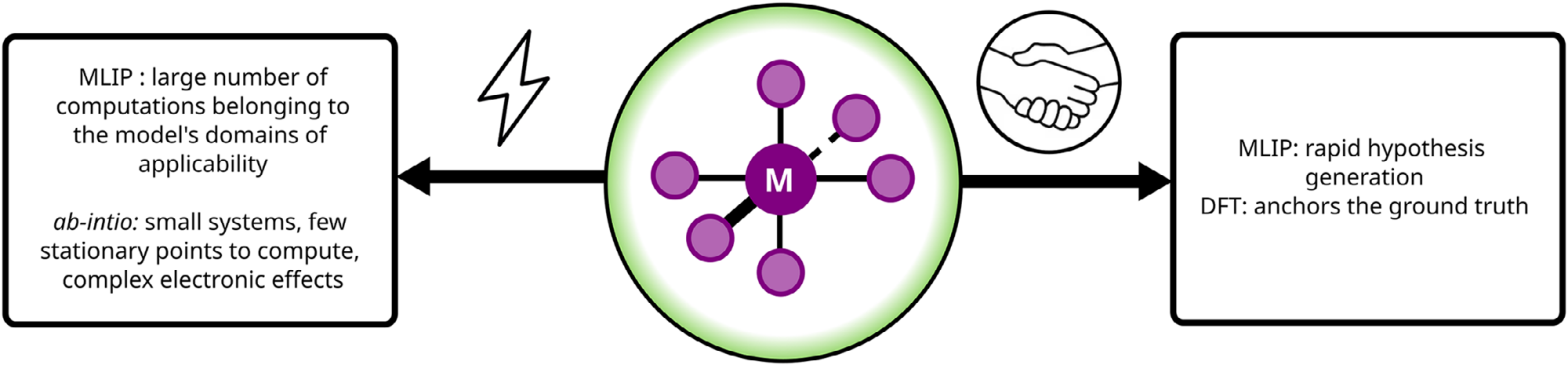
Domains where ML potentials can credibly replace routine ab‐initio calculations and where a synergy is inevitable.

Competition is less compelling when a catalytic conclusion depends on small energy differences driven by electronic effects rather than broad geometric trends. In homogeneous catalysis, selectivity and turnover often depend on barrier differences of only a few kcal ${\mathrm{mol}}^{-1}$. Foundation MLIPs primarily learn a mapping from atomic structure, and sometimes global labels such as charge and spin, to energies and forces. They therefore rarely provide the electronic diagnostics used to judge whether an energy difference is meaningful. Electronically delicate steps are thus where ML speed should be paired with electronic‐structure adjudication rather than used as a drop‐in replacement (Figure 3).

This does not make MLIPs irrelevant in high‐stakes regions. When the target chemistry lies well within the pretraining manifold, such as common coordination motifs in closed‐shell complexes with familiar ligands and charge states, modern MLIPs often reproduce structures and relative energetics well enough to guide exploration and generate hypotheses. However, when mechanisms involve unusual oxidation states, low coordination numbers, pronounced multireference character, explicit charge transfer, or competing spin manifolds, the model prior is often insufficient, and errors become systematic. In these cases, even a plausible ML trajectory may be misleading if the electronic state is incorrect. For mechanistic claims, ML‐driven exploration should therefore be coupled to escalation to DFT and higher‐level methods. A key difference is that DFT provides diagnostics rooted in physics (e.g., the existence of near‐degenerate states or orbitals) that suggest a need for escalation to higher‐level methods to knowledgeable practitioners. An analogous diagnosis is not trivial for MLIPs. We return to the hardest electronic regimes and DFT limitations in Section 7.

Synergy is also pragmatic. As systems get smaller and the number of decisive stationary points shrinks, the raw speed advantage of MLIPs matters less. For many homogeneous catalysts with tens of atoms, a modest set of DFT calculations on selectivity‐determining steps is already feasible. In that setting, replacing final adjudication with ML adds risk without proportional benefit. The cost landscape is also evolving because GPU‐optimized electronic‐structure implementations continue to reduce DFT wall times, narrowing the gap for the few computations that decide a mechanism [56, 57].

The most robust near‐term strategy is a clear division of labor. MLIPs compete where the goal is breadth. They generate ensembles, map reaction space, and surface candidate structures quickly and cheaply. DFT anchors the points where electronic structure decides the answer and connects with higher‐level methods if required. In practice, ML outputs should be treated as high‐coverage proposals, while electronic‐structure methods are reserved for verification and refinement whenever conclusions require fine energetic discrimination or correct state identity. With explicit escalation criteria, this hybrid approach preserves ML throughput gains without sacrificing the diagnostic richness and mechanistic rigor that homogeneous catalysis demands.

## 5 Reliability, Uncertainty, and Escalation

In homogeneous catalysis, speed matters only when predictions are trustworthy. For foundation MLIPs, trust is not captured by a single global MAE or RMSE on random train and test splits because real workflows generate related configurations rather than independent ones. This is most obvious near bond rearrangements, unusual coordination changes, and strained geometries. In practice, a workflow needs a warning signal for each structure that indicates when a predicted energy or force is likely to be wrong. This is the role of uncertainty quantification (UQ). The goal is a per‐structure estimate of how reliable the predicted energies and forces are, together with evidence that the stated confidence matches the observed error for the deployment domain, including metals, ligands, charge and spin states, and solvation motifs [34, 35].

**FIGURE 4:**
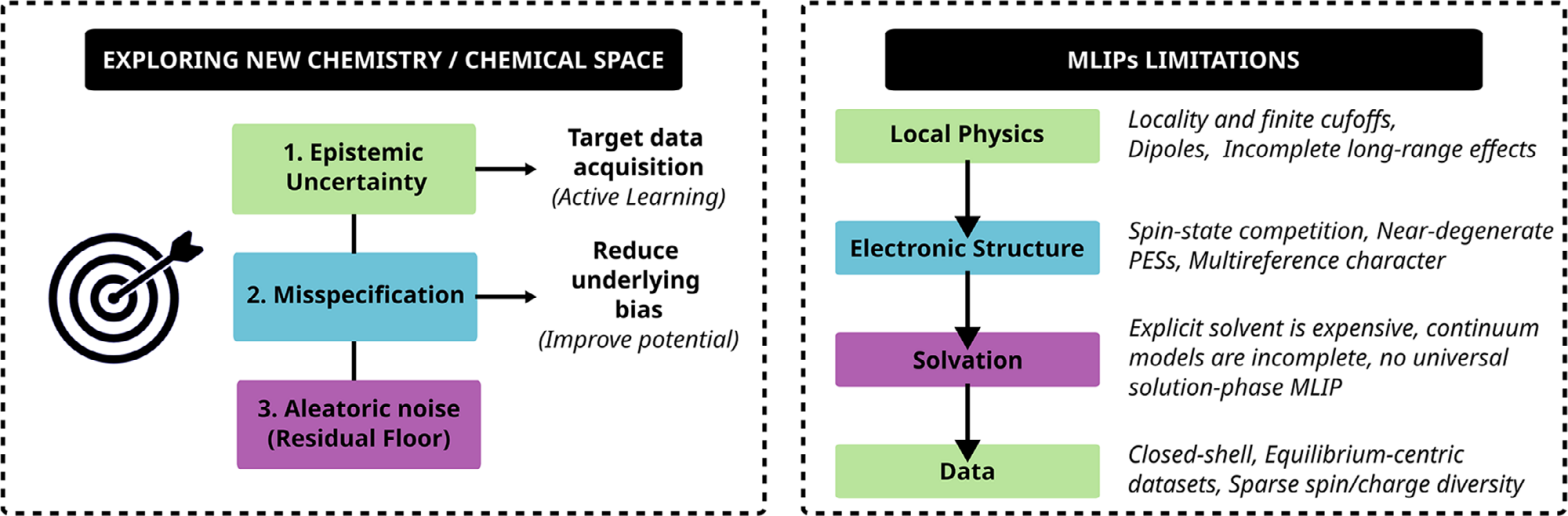
Reliability, uncertainty quantification, and limitations in ML‐driven workflows applied to homogeneous catalysis.

It helps to separate three sources of uncertainty that behave differently in catalytic workflows (Figure 4). *Aleatoric* uncertainty reflects noise or variability in the reference labels. For foundation MLIPs, the labels are typically DFT energies and forces, so this uncertainty includes the intrinsic scatter and approximations of the chosen DFT protocol. It sets an error floor that no model trained on those labels will systematically beat, and it is best addressed during training with losses that allow structure‐dependent noise [34]. *Epistemic* uncertainty reflects lack of coverage. It rises when the model sees chemistries or geometries that differ from what it was trained on, such as new ligand classes, uncommon coordination motifs, changes in oxidation or spin state, rare conformations, or distorted structures near reactive events. Epistemic uncertainty decreases when informative new reference calculations are added. In deployed models, it is often estimated by checking how sensitive the prediction is to small changes in the model, for example, by comparing several independently trained models or by using dropout at prediction time [34, 35]. *Misspecification* arises when the model architecture lacks important physics. In that case, a model may give confident‐looking predictions that are systematically biased, for example, when long‐range electrostatics and polarization, solvation effects, or spin and charge phenomena are represented poorly [58, 59, 60]. In catalysis, epistemic uncertainty often dominates early exploration, while misspecification becomes limiting as coverage increases since better uncertainty estimates do not fix missing physics [58, 59].

While capturing epistemic uncertainty is already an established part of MLIP workflows, it is typically achieved through model ensembles [42]. This has important drawbacks in terms of cost, since several models must be trained and used to predict at each step. Alternatively, there, approaches such as evidential learning, where a single network predicts not only an energy or force but also a distribution that represents confidence, are attracting interest [61]. In molecular property prediction, evidential methods often produce uncertainties that track error and guide data selection at essentially no added prediction cost, which suggests a promising direction for atomistic models when running many models is too expensive.

Because catalytic conclusions often hinge on small energy differences, UQ must be meaningful at the level of decisions. The relevant target is not only uncertainty in an absolute energy, but also uncertainty in barrier differences, free‐energy spans, and ensemble‐averaged free energies used in microkinetic models [62, 63]. This makes calibration essential. An uncertainty estimate is useful only if, on average, it matches real errors. Raw uncertainties from models are rarely calibrated out of the box, especially under distribution shift, so deployment should include calibration on a small, domain‐matched reference set that reflects the configurations the workflow will generate [34]. Practically, one defines the deployment domain and the reference level of theory, builds a calibration set that mirrors typical structures including relevant distortions and solvent or ion motifs, and rescales the raw uncertainty so that stated confidence matches observed error. Post‐hoc scaling and conformal approaches are attractive because they are simple and robust, and they provide reliable coverage under mild shifts, particularly when conditioned on whether a structure looks familiar in the model's internal representation [34, 64]. Calibration should be checked both in‐domain and on targeted stress tests that mimic catalytic failure modes [65].

Even with calibration, failures are inevitable, so the objective is early detection and selective escalation rather than unconditional trust. In an MLIP‐driven workflow, monitoring can be embedded into optimization, sampling, and pathway exploration. ML steps are accepted while uncertainty stays within a predefined envelope, and escalation is triggered when uncertainty rises sharply or when stability diagnostics indicate extrapolation. Practical indicators include unusually large predicted forces, sudden energy changes along short steps, repeated optimization failures, persistent nonconvergence, or geometries that are chemically implausible [65, 66, 67]. Flagged configurations are then refined with electronic‐structure calculations, typically DFT and sometimes higher‐level methods for electronically delicate cases, and the workflow continues using validated results. This escalation loop is central to safe use because it makes ML speed conditional on reliability and routes difficult cases to methods that provide electronic diagnostics and controlled accuracy [53, 67, 68, 69].

A tension remains between what is ideal for model improvement and what is convenient for practitioners. In bespoke MLIP development, escalation points become new training data in active‐learning cycles. For off‐the‐shelf foundation MLIPs, fine‐tuning is often impractical for nonexperts, so escalation may simply mean switching to DFT for problematic segments of a workflow. Even so, flagged structures and validated reference calculations remain valuable. If captured with sufficient metadata and shared in reusable form, they become targeted additions to the next iteration of foundation training corpora and benchmarks, turning deployment failures into systematic coverage expansion (Section 8) [70, 71].

Finally, multi‐fidelity strategies offer a complementary route to reliability by separating broad coverage from high‐stakes accuracy. Rather than training one potential that is uniformly high fidelity, a practical approach uses abundant DFT‐level data for breadth and adds sparse, carefully selected higher‐level corrections via Δ‐learning on chemically decisive subsets [72]. In catalysis, these subsets include transition‐state neighborhoods, spin‐state splittings, redox‐active motifs, and other regions where small errors have outsized mechanistic impact. The open question is not whether multi‐fidelity helps, but how best to integrate it so escalation, calibration, and fidelity selection become routine and standardized rather than ad hoc.

## 6 Closing the Gaps in ML Potentials

Foundation‐level MLIPs trained across molecules, materials, and catalysis inherit a central limitation of many modern architectures: they are largely local models with finite cutoffs, so performance drops when nonlocal physics controls energies and forces (Figure 4). In homogeneous catalysis this is common, since solution‐phase reactivity and charged or open‐shell organometallics are often governed by long‐range electrostatics, environment‐dependent polarization, and non‐local charge transfer. Purely local representations struggle to reproduce dielectric screening, field response, and electron redistribution across extended ligand frameworks. Errors may look modest near equilibrium yet become consequential along distorted reaction coordinates, where dipoles, solvation energetics, and barrier heights depend on physics outside a short‐range neighborhood [59]. While in some condensed phases long‐range information may be transferred through geometric effects and successfully captured by the MLIPs [73, 74], ultimately these are misspecification problems due to local architectures. Even perfect coverage of local geometries does not guarantee a correct response to long‐range perturbations [23, 24].

A practical remedy is to augment a short‐range MLIP with a self‐consistent charge‐equilibration layer, such as QEq or kQEq. These models predict environment‐dependent electronegativities and solve for a global charge distribution at each step, yielding long‐range electrostatics and an implicit form of polarization that improves dipoles, forces, and transferability for ionic and polar systems [75, 76]. However, naive charge equilibration may overpolarize or induce unphysical charge transfer, especially in heterogeneous environments, so successful use typically requires regularization and physically motivated constraints [77]. The broader message is that long‐range behavior is rarely learned by scaling local data alone.

Complementary strategies add explicit long‐range terms while leaving the MLIP to model chemically complex short‐range interactions. One pattern constructs Coulomb interactions from analytic charge distributions, for example associated with ions and Wannier centers, while a neural potential models the short‐range remainder, which restores correct tails and improves transfer to larger systems [78]. Another approach treats London dispersion explicitly by learning atomic dispersion coefficients and evaluating the asymptotic term, which improves intermolecular interactions and mitigates a known weakness of purely local regression [79]. These add‐ons are attractive because they are orthogonal to dataset scale, and enforcing correct physical limits reduces the burden on the network and improves extrapolation. For a recent perspective on long‐range electrostatics for MLIPs, we refer the reader elsewhere [80].

Architectural routes to nonlocality are advancing in parallel. Long‐range‐aware message passing, attention‐based designs, and hybrids with analytic corrections propagate information across a structure more effectively than fixed‐cutoff schemes. Some architectures also incorporate charge and spin conditioning to improve state awareness [46, 81]. The likely near‐term outcome is not a single winner but robust stacks that combine a strong short‐range backbone with explicit electrostatics and polarization and long‐range‐aware components, tuned to dominant failure modes in the target domain [23, 24, 59, 82].

Solvation compounds these challenges because realistic solution‐phase catalysis requires accurate energetics and extensive sampling. Continuum models often miss specific hydrogen bonding, entropic pre‐organization, and solvent‐mediated charge rearrangements, while explicit solvent introduces large configurational spaces and rare‐event sampling barriers. Here MLIPs offer a real opportunity. Low‐cost forces make explicit‐solvent sampling, enhanced sampling, and larger ensembles practical. Recent demonstrations show that, with active learning, explicit‐solvent ML potentials reproduce liquid structure and deliver adsorption and free‐energy quantities with near‐DFT fidelity at much lower cost [42, 44, 83]. Nonetheless, condensed‐phase catalysis remains difficult because pathway exploration is combinatorial, sampling is expensive, and charged spectators such as counterions interact through long‐range physics that local models treat poorly [43, 84, 85, 86].

Implicit solvation coupled to MLIPs is less mature. A general strategy would mirror DFT practice by combining a transferable gas‐phase MLIP with an implicit solvent model. This adds requirements. The MLIP must provide reliable charges and multipoles, or an equivalent electrostatic representation, so the solvation model responds correctly across conformations and charge states. ML‐augmented continuum approaches, such as ML‐corrected PCM, show that residual learning improves solvation free energies when explicit solvent is infeasible [87], but broadly usable, foundation‐level solutions that make off‐the‐shelf MLIPs reliable for solution‐phase catalysis are not yet available. Until this gap closes, solvation remains a bottleneck for deploying foundation MLIPs as default engines for homogeneous catalysis rather than fast scouts [88, 89].

**FIGURE 5:**
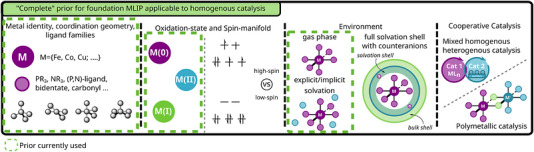
Schematic representation of what would constitute a “complete” prior for the development of a foundation MLIP applicable to homogeneous catalysts, and the current state.

In summary, credible competition with DFT for solution‐phase catalysis, charged complexes, and open‐shell intermediates requires explicit treatment of long‐range electrostatics and polarization and a practical solvation strategy (Figure 5). The most robust near‐term stacks will combine a strong short‐range MLIP with self‐consistent electrostatics, such as charge equilibration, explicit long‐range corrections for electrostatics and dispersion, and long‐range‐aware architectures, validated under the sampling conditions that catalysis demands [23, 24, 59].

## 7 The Hard Boundaries

Homogeneous catalysis often operates in an electronic regime where several configurations lie close in energy. First‐row transition metals, open‐shell intermediates, variable ligand fields, and bond‐making and bond‐breaking transition states naturally generate near‐degeneracies and competing spin manifolds. In these systems, the mechanistically decisive object is often not a single potential energy surface (PES), but the relative placement and coupling of several PESs. Errors of only a few kcal ${\mathrm{mol}}^{-1}$ may reorder spin ladders, shift crossing points, and invert predicted selectivity [13]. This sets a boundary condition for any surrogate that maps structure to energy. When the identity of the relevant electronic state changes along the reaction coordinate, the energy is no longer a single‐valued function of geometry unless the state is specified and tracked.

For foundation MLIPs, this issue is acute because most models predict energies and forces from atomic positions, and sometimes global labels such as total charge and spin, without explicit local electronic degrees of freedom. Early generations were effectively insensitive to electron count and spin, which limited use whenever multiple charge or spin surfaces matter, including in redox chemistry [40]. More recent architectures and datasets incorporate total charge and spin information, which is necessary, but it does not resolve the deeper ambiguity that arises when multiple electronic configurations with the same total charge and spin coexist at similar geometries and differ mainly by orbital occupations. In such cases, the mapping from structure to energy becomes effectively multivalued unless the model is conditioned on a richer state description or trained explicitly on multi‐state data. Spin‐crossover systems and other near‐degenerate manifolds, as encountered in many iron and cobalt catalysts with competing high‐spin and low‐spin pathways, therefore remain difficult for current MLIPs to treat reliably. Related issues appear in redox‐active ligands and ligand‐to‐metal charge‐transfer motifs, where small structural changes drive large redistributions of electron density and polarization that are not straightforwardly encoded by local geometric descriptors. Mechanistic conclusions in these regimes depend on electronic diagnostics such as spin densities, charge localization, and orbital occupations, which are native to electronic‐structure methods but largely absent from standard MLIP outputs.

This is also where DFT itself becomes a moving target rather than a fixed reference. Spin‐state energetics are strongly method‐dependent, and credible, broadly applicable reference data are scarce, so reasonable DFT protocols may disagree qualitatively for the same complex [13, 90]. The SSE17 benchmark set, derived from experimental spin‐state splittings, illustrates the magnitude of the challenge. Common hybrid and meta‐hybrid functionals often show mean absolute errors of about 5–7 kcal ${\mathrm{mol}}^{-1}$ and maximum errors above 10 kcal ${\mathrm{mol}}^{-1}$. Double hybrids reduce errors, and CCSD(T) approaches chemical accuracy on that set [13]. Even higher‐level approaches may remain inconsistent for open‐shell transition‐metal energetics unless protocols are tightly controlled. The limitation is not simply that DFT fails, but that the electronic structure is intrinsically hard [11, 12, 13, 90, 91]. When an MLIP is trained on DFT, it may inherit systematic bias in the reference. When DFT itself is uncertain, ML will not surpass it without targeted higher‐level data and careful multi‐fidelity design.

Wavefunction methods provide the principled route forward because they represent strong correlation beyond DFT's mean‐field framework, but routine use in catalytic workflows remains difficult due to scaling, convergence sensitivity, and the practical reality that multireference and coupled‐cluster energetics are often evaluated as single points on DFT geometries rather than through full geometry optimization [90]. A similar boundary appears in photocatalysis and other excited‐state mechanisms. The relevant landscape involves multiple electronic states and nonadiabatic couplings, and even within electronic‐structure theory the balance between accuracy, robustness, and cost becomes delicate. For MLIPs, the implication is direct. Unless models are trained on multi‐state data and equipped to represent state identity and couplings, they should not be expected to reproduce crossings, reordering, or state‐specific barrier heights in a reliable and transferable way [92].

A realistic near‐term role for ML in these boundary regimes is assisted exploration paired with electronic‐structure diagnostics rather than autonomous prediction. Practically, this means using MLIPs to accelerate candidate geometry generation and sampling where they remain stable, while prioritizing escalation to DFT and higher‐level methods at points where state competition is expected or where small energetic differences control conclusions. Recent analyses suggest that differences in multireference character between states or structures, often termed multireference imbalance, rather than absolute multireference character alone, are often more predictive of property errors. This provides a principled criterion for where to invest higher‐level calculations within multi‐level workflows [93]. In the longer term, progress toward foundation MLIPs that remain reliable across strongly correlated regimes will require larger datasets and procedural advances that make higher‐level data generation more robust and scalable. Even aside from computational cost, the human and algorithmic burden of generating converged multireference labels for diverse, out‐of‐equilibrium structures is far higher than for DFT. Progress will likely rely on improved automation, better diagnostics, and multi‐fidelity strategies that introduce sparse high‐level corrections only where they change decisions [94, 95, 96, 97].

In short, the hard boundaries for foundation MLIPs in homogeneous catalysis are defined by state competition and strong correlation. These are situations where the reaction coordinate traverses multiple electronic surfaces, where spin and charge localization change abruptly, or where mechanistic conclusions rely on electronic diagnostics rather than geometry alone. These regimes do not eliminate the value of MLIPs, but they enforce synergy by construction. ML accelerates exploration, while electronic‐structure methods remain essential to identify the correct state, quantify decisive energy differences, and provide the diagnostics needed for mechanistic credibility.

## 8 Data and FAIR Standards

In spite of the previous sections, the prospects of foundation MLIPs in homogeneous catalysis probably depend less on model class than on the availability of reference data. Key needs are chemically diverse transition‐metal and ligand environments, off‐equilibrium geometries with forces, and explicit total charge and spin multiplicity [46, 98]. Unlike biomolecular MLIP domains, homogeneous catalysis routinely spans multiple oxidation states, spin manifolds, and coordination topologies, which increases the demands on coverage and electronic‐state labeling [46, 82, 99, 100, 101, 102, 103].

Because modeling must span several potential energy surfaces across the periodic table, chemical space will not be covered by enumeration, which reaches extraordinary scales even for small organic molecules alone [104]. For MLIPs, useful coverage is instead the diversity of local atomic environments and electronic states visited by simulations. This motivates catalysis benchmarks organized around motifs that matter for dynamics and reactivity, such as coordination changes, bond‐making and bond‐breaking neighborhoods, and charge and spin variation, rather than static collections of optimized structures alone [105, 106]. Recent benchmarking discussions likewise argue that dataset design and sampling of relevant dynamical modes often dominate downstream simulation reliability beyond what energy and force errors alone suggest [53, 107]. Because molecular MLIP datasets often contain strongly correlated configurations, such as trajectory frames or small distortions, random splits may substantially overestimate generalization.

Transition‐metal complexes still lag behind organic molecules in data availability, and large corpora remain essential anchors for representation learning and baseline evaluation. The pioneering tmQM dataset provides tens of thousands of mononuclear transition‐metal complexes mined from the Cambridge Structural Database (CSD) and labeled with computed properties at standardized levels of theory [108]. Recomputations and curated variants such as tmQM_wB97MV address inconsistencies and improve usability [109]. Label‐enriched extensions push beyond geometry‐only learning. tmQMg supplies NBO‐informed natural quantum graphs (NatQG) for a large subset, enabling models to exploit chemically meaningful electronic descriptors [110], and tmQM+ augments tmQM with descriptors at multiple levels of theory to probe robustness and transfer to unseen regimes [111]. Application‐linked curation, exemplified by tmCAT, increases catalytic relevance by identifying catalysis‐associated subsets from broad transition‐metal databases [112]. Crystallography‐mining tools such as cell2mol recover connectivity and total charge, including oxidation state, from experimental structures, which helps bridge the CSD to usable datasets [113, 114]. Complementary developments that infer ground‐state spin directly from structure, such as TM‐GSspin, address another frequently missing label for charge‐ and spin‐aware modeling [115].

For catalysis‐facing applications, labels beyond energies and forces are valuable as state descriptors and diagnostics. Consistent total charge and spin, atomic charge and spin populations, dipoles and multipoles, bond‐order proxies, or density‐derived descriptors help define what is being modeled and enable like‐for‐like comparisons across datasets and levels of theory [99, 108, 110, 111, 116]. This is already visible in molecular datasets that intentionally expose richer label spaces [99, 108].

For MLIPs, the central gap is also nonequilibrium coverage, meaning forces on distorted geometries resembling what trajectories explore. The original ANI‐1 dataset illustrated the importance of large‐scale off‐equilibrium sampling for molecular MLIPs [117], and in organic chemistry the Transition1x database shows how pathway‐adjacent sampling can be systematized at scale using NEB‐generated reaction paths [106]. Active‐learning workflows likewise show that model‐guided selection broadens coverage with fewer redundant points than naive sampling, as in ANI‐1x [105, 118]. Benchmarks should include activated or rare‐event configurations, such as transition‐state neighborhoods and strained intermediates, since these can control kinetics yet remain poorly learned even when average force and energy errors are low [53, 106]. In practice, stable long‐time trajectories may fail despite low test‐set errors, often reflecting excursions into poorly covered regions of configuration space [119]. For homogeneous catalysis, analogous benchmarks should prioritize strained coordination geometries and reaction‐center distortions characteristic of organometallic mechanisms, since these regions are sparsely sampled by equilibrium‐focused corpora yet disproportionately important for reactivity [101, 120].

A second constraint is accuracy. Obtaining training data beyond DFT requires multi‐fidelity reference strategies. The realistic path is not coupled‐cluster calculations everywhere, but carefully designed mixtures of levels of theory. Transfer learning shows that a model pretrained on abundant DFT data can be elevated toward coupled‐cluster quality with a comparatively small, carefully selected high‐level subset [118, 121]. A complementary route combines a fast baseline quantum method with learned corrections, exemplified by AIQM1 [122]. Future trends may retain DFT for breadth, including conformers, solvent motifs, and coordination variants, while concentrating higher‐level calculations on chemically decisive regions, such as transition states, spin‐state splittings, and redox‐active motifs, where marginal accuracy gains matter most [111, 121]. Even if multi‐fidelity approaches reduce the need for extensive coupled‐cluster and multireference data, improving the quality and accessibility of such computations remains critical for reliability in the difficult regimes most relevant to catalysis.

**FIGURE 6:**
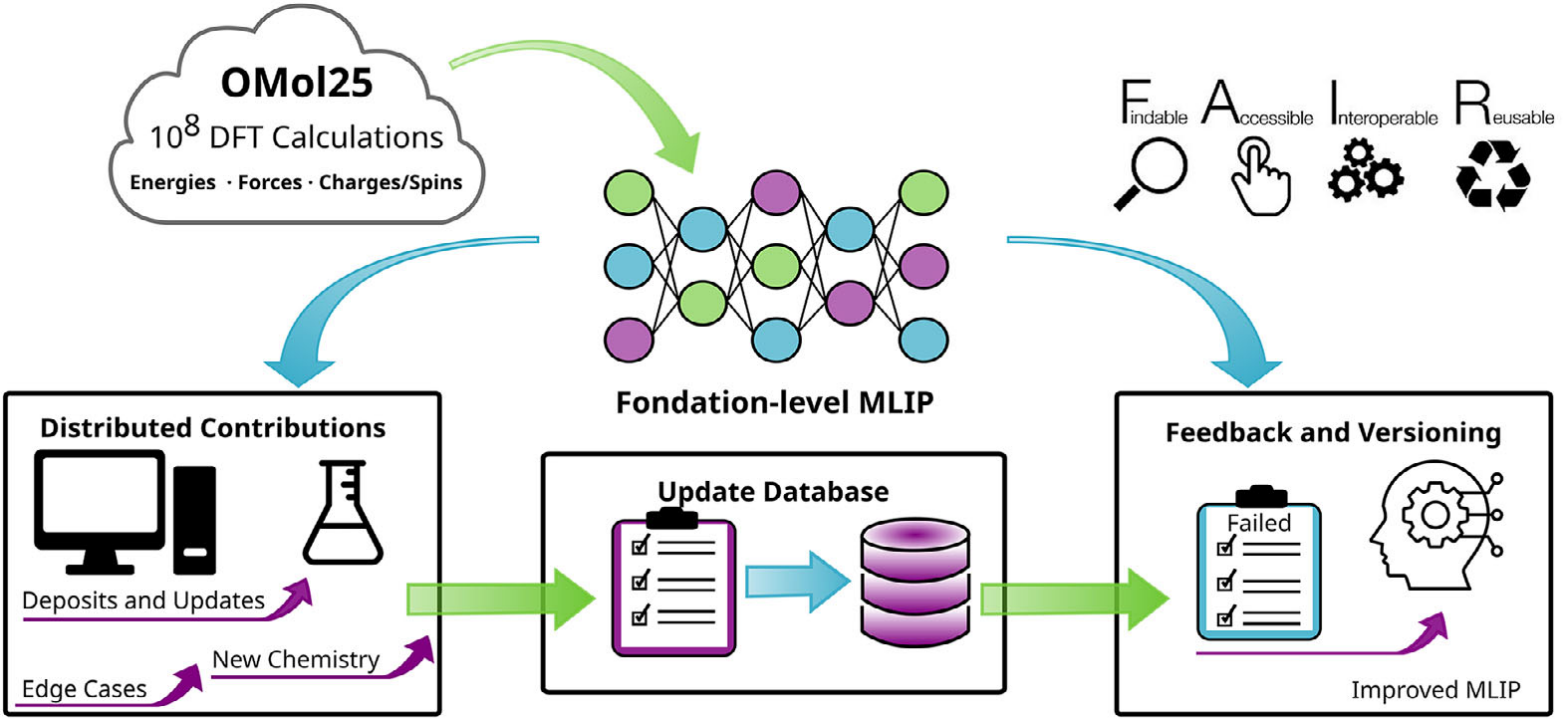
Schematic representation of a potential future for FAIR data infrastructures, illustrated using OMol as an example.

In parallel, aggregating foundation corpora recomputed at a consistent level of theory reduces friction in cross‐domain training and benchmarking. OMol25 provides $>10^{8}$ DFT calculations at a uniform hybrid‐DFT level, including energies, forces, and explicit storage of charge and spin multiplicity, spanning broad molecular chemistry and including metal complexes [47, 48]. Efforts at this scale align levels of theory and metadata across domains, but they may be complemented by continuous, distributed growth in which new chemistries and failure cases are deposited, versioned, and merged without an all‐at‐once campaign [47, 48, 123, 124]. Because a foundation model is attractive precisely for out‐of‐the‐box use, a key open question is how practitioners will communicate failures and share escalated DFT data back to providers in reusable, versioned form. Closing this loop would enable a community‐driven, continuous improvement of the MLIPs (Figure 6) [119, 125].

Finally, the difference between a large dataset and a usable benchmark increasingly hinges on FAIR implementation, including persistent identifiers, machine‐readable schemas, complete provenance, and licenses that permit community evaluation. ioChem‐BD enables curated deposition and publication of computational chemistry data with provenance and DOIs [126], QCArchive provides an API‐first platform and standardized schemas for large‐scale quantum‐chemistry campaigns and programmatic reuse [124], and meta‐layer efforts such as OpenQDC and ColabFit Exchange consolidate heterogeneous quantum‐mechanical datasets into standardized, ML‐ready formats for cross‐dataset benchmarking [123, 127]. Recent surveys of quantum‐chemical datasets and databases for ML potentials further emphasize the need for sustainable, updatable resources and standardization alongside FAIR alignment [128]. For catalysis‐facing MLIP benchmarks, a minimal FAIR checklist should include explicit total charge and spin multiplicity, energies and per‐atom forces with units, unambiguous solvation and counterion conventions, complete provenance, including code and version, functional, basis, dispersion, and thresholds, and documented curation steps covering duplicates, outliers, and structure hygiene [123, 124, 126].

Taken together, the near‐term opportunity is not a single universal catalysis dataset, but an ecosystem of versioned, interoperable benchmarks. These benchmarks should expand through targeted additions when models encounter new ligand, metal, or oxidation‐state regimes, embed multi‐fidelity upgrades where DFT is most fragile, and make charge, spin, and diagnostically useful auxiliary properties explicit by design [47, 101, 112, 121].

## 9 Conclusion

Foundation machine learning potentials hold the potential to shift homogeneous catalysis modeling from a regime limited by individual electronic structure evaluations to one limited by sampling design, decision thresholds, and traceable provenance. Over the next few years, the most visible disruption will occur in the exploratory layers of a catalysis workflow. Conformer and speciation enumeration, reaction network scouting, and transition state candidate generation often demand orders of magnitude more structures than the final mechanistic narrative ever reports. In that breadth‐first setting, pretrained potentials will increasingly serve as default engines for producing high‐coverage proposals, while routine low‐level DFT steps and semiempirical methods recede.

Competition will feel credible only when it is paired with reliability. Applicability domains should be declared up front, and uncertainty estimates, even when approximate, must be calibrated on domain‐matched distortions and tied to automatic escalation. Just as importantly, every escalation should leave a reusable footprint through versioned structures, methods, metadata, and outcomes that flow back into community benchmarks and future foundation corpora. Long‐term impact will hinge less on scaling model size and more on closing the physics and data gaps that dominate solution phase organometallic chemistry. Multi‐fidelity strategies will mature into shared infrastructure. Delta learning libraries targeting common organometallic motifs and reactions may become a valuable resource for fine‐tuning and adaptation.

The strongest signal of synergy will be end‐to‐end exemplars that connect simulation to experiment. ML potentials generate ensembles and solvent‐conditioned free energies, electronic structure methods diagnose state identity and refine decisive steps, and microkinetic models propagate those inputs into rates and selectivities with quantified uncertainty. As these pipelines become reproducible, portable, and benchmarked on failure‐focused stress tests, we may stop debating replacement and instead treat ML and electronic structure as coupled layers in a single predictive stack.

## Conflicts of Interest

The authors declare no conflicts of interest.
